# Correlation between Gensini Score and Duration of Diabetes in Patients Undergoing Coronary Angiography

**DOI:** 10.7759/cureus.4010

**Published:** 2019-02-04

**Authors:** Razi Ul Amin, Muhammad Anis M Ahmedani, Musa Karim, Ahmed Raheem

**Affiliations:** 1 Cardiology, National Institute of Cardiovascular Diseases, Karachi, PAK; 2 Miscellaneous, National Institute of Cardiovascular Diseases, Karachi, PAK; 3 Pathology, Aga Khan University Hospital, Karachi, PAK

**Keywords:** diabetes, gensini score, duration, angiography, coronary artery disease

## Abstract

Introduction

The relationship between the duration of diabetes mellitus and coronary artery disease (CAD) is well established. Moreover, the Gensini score system is a reliable assessment tool for the severity of coronary artery disease (CAD). After an extensive literature search, we found that there is a knowledge gap about the relationship between the Gensini score and the duration of diabetes in our population. Therefore, the aim of this study was to find the relationship between the Gensini score and the duration of diabetes in patients undergoing a coronary angiography.

Methods

A cross-sectional study was conducted among 321 consecutively selected diabetic patients. Clinically diagnosed cases of diabetes on proper anti-diabetic treatment were included in this study. Patients with known severe CAD or history of coronary artery bypass grafting (CABG) surgery or primary coronary intervention (PCI) were excluded from the study. Coronary angiography was performed on all the patients and their Gensini score was calculated using the modified scoring schema. Duration of diabetes and other baseline risk factors were recorded for all patients. The relationship between the Gensini score and the duration of diabetes was assessed by calculating Pearson’s correlation coefficient.

Results

A total of 321 diabetic patients were included in this study, out of which 67.9% (218) were men and mean ± standard deviation age was 56.13 ± 7.67 years ranging between 40 to 70 years with a majority of the patients, 63.9% (205), being under 60 years of age. Along with diabetes, the most commonly observed risk factor was hypertension, which was observed in 77.6% (249) of the patients. Smoking and obesity were also observed in 18.7% (60) and 17.4% (56) of the patients, respectively. A significant positive correlation, 0.55 (p<0.001), was observed between the duration of diabetes and the Gensini scores. The correlation was found to be stronger in older patients (more than 60 years of age) with a correlation coefficient of 0.52 vs. 0.38, and male patients with correlation coefficients of 0.66 vs. 0.34.

Conclusion

A significant positive correlation between the Gensini score and the duration of diabetes mellitus was observed. This correlation is relatively stronger among male and older patients (more than 60 years of age).

## Introduction

Atherosclerosis in coronary arteries cause coronary artery disease (CAD), which is the cause behind most of the mortality as well as morbidity around the globe. Detection of atherosclerosis could be possible in its earlier subclinical asymptomatic stage, which gradually develops into the clinically overt disease with the passage of time [1-2]. Cardiovascular diseases have emerged as a major public health problem over the last couple of decades not only in developed countries but also in developing countries [3-4]. By 2020, annual deaths due to CAD is projected to rise to 11.1 million in developing countries, and mortalities due to coronary artery disease in 2020 are projected to rise by 137% among men and 120% among women as compared to 1990 in developing countries [5-6]. With the passage of time, diabetes mellitus (DM) is becoming an epidemic around the world; by 2030, the number of diabetic patients will probably rise to 366 million from 171 million in the year 2000 [7]. A large part of this increase is expected to derive from low-income, underdeveloped, and developing countries such as South Asian countries, Sub-African countries, Latin America, and some parts of the Middle East [8].

In Pakistan, the number of people having DM is almost the same in both rural and urban areas, with 9.4% of the population in rural areas being affected by DM and 9.5% in urban areas [9]. The World Health Organization (WHO) ranked Pakistan as the seventh most affected country by diabetes. However, these numbers may still be underreported, as there are many cases that go unreported or undiagnosed [9-10]. The high prevalence of diabetes, and the yearly increase in the number of reported cases, particularly of type two, in Pakistan poses not only a threat to the overall economy of the country, but also results in a poorer quality of life due to the high costs associated with care coupled with high rates of complications due to poor glycemic control [11].

Insulin resistance appears to be associated with CAD and hyperinsulinemia and is a positive free hazard for coronary heart disease (CHD). Additionally, insulin resistance adds to the advancement of cardiovascular risk factors [12]. It was proposed that the measurement of insulin resistance be added as a dependent of the severity of coronary artery disease (CAD) and more severe, multi-vessel, and long segment CAD was found in patients with diabetes lasting for more than five years as compared to those patients who had had diabetes for a duration of less than or equal to five years [13]. The Gensini score system is a technique developed by Gensini et al. [14], for the assessment of the severity of coronary artery disease (CAD). This scoring system is based on the artery morphology, coronary anatomy, and severity of stenosis in lesions [15]. A strong association of Gensini score was observed with long and short-term cardiovascular risk [16].

After an extensive literature search, only a few studies were found to have been published in our population that had studied the relationship between their Gensini score and the duration of diabetes. It is important to explore this relationship in our local population so that better management, counseling, and preventive strategies towards the risk reduction of CAD can be formulated for this subgroup of our population. Therefore, the aim of this study was to find the relationship between the Gensini score and the duration of diabetes in patients undergoing coronary angiography.

## Materials and methods

This study was carried out in the outpatient department of the National Institute of Cardiovascular Diseases (NICVD), after receiving approval from the ethics committee of NICVD (ERC-21/2018). The participants were fully informed about the study and written consent was obtained before their enrollment. Clinically diagnosed cases of diabetes on proper anti-diabetic treatment were included in this study. Patients with known severe CAD or prior history of coronary artery bypass grafting (CABG) surgery or primary coronary intervention (PCI) were excluded from the study. The coronary angiography was performed and interpreted by an interventional cardiologist who has more than five years of working experience. A modified Gensini score was calculated using the scoring schema defined by Gensini et al. [14]. In order to avoid observation bias, the Gensini scores were calculated by three independent cardiologists and a round-up of an average of the three was considered for the final analysis. The duration of diabetes was recorded in roundup years since the clinical confirmation of diabetes mellitus. Clinical characteristics such as hypertension (on antihypertensive medication for at least six months), obesity (calculated body mass index > 30 kg/m^2^), and smoking (history of smoking in past one year) were obtained. All demographic and study variables were imported into SPSS Statistics for Windows, Version 21.0 (IBM Corp., Armonk, NY, US) and R software version 3.5.1 (The R Foundation for Statistical Computing). The Kolmogorov-Smirnov (KS) test was applied to the check the hypothesis of normality of distribution for both the study variables: duration of diabetes (years) and Gensini score. Mean ± standard deviation (SD) was calculated and the two subgroups were compared by applying the Mann-Whitney U test. The relationship between the Gensini score and the duration of diabetes was assessed by calculating the Pearson’s correlation. In addition, a significance criterion was set as p-value equals or below 0.05.

## Results

A total of 321 diabetic patients were included in this study, out of which 67.9% (218) were men and mean ± standard deviation age was 56.13 ± 7.67 years ranging between 40 to 70 years with a majority of the patients, 63.9% (205), being under 60 years of age. Along with diabetes, the most commonly observed risk factor was hypertension, which was observed in 77.6% (249) patients. Smoking and obesity were observed in 18.7% (60) and 17.4% (56) patients, respectively. Mean body mass index (BMI) was calculated to be 26.53 ± 4.38 kg/m^2^. Baseline demographic characteristics are presented in Table 1.

**Table 1:** Baseline demographic characteristics

Characteristics	Total (n = 321)
Gender	
Female	103 [32.1%]
Male	218 [67.9%]
Age	56.13 ± 7.67 years
≤ 60 years	205 [63.9%]
> 60 years	116 [36.1%]
Risk profile	
Hypertension	249 [77.6%]
Smoking	60 [18.7%]
Obesity	56 [17.4%]
Body mass index (BMI)	26.53 ± 4.38 kg/m^2^
Duration of diabetes	12.46 ± 4.86 years
Gensini score	71.99 ± 44.12

**Figure 1:**
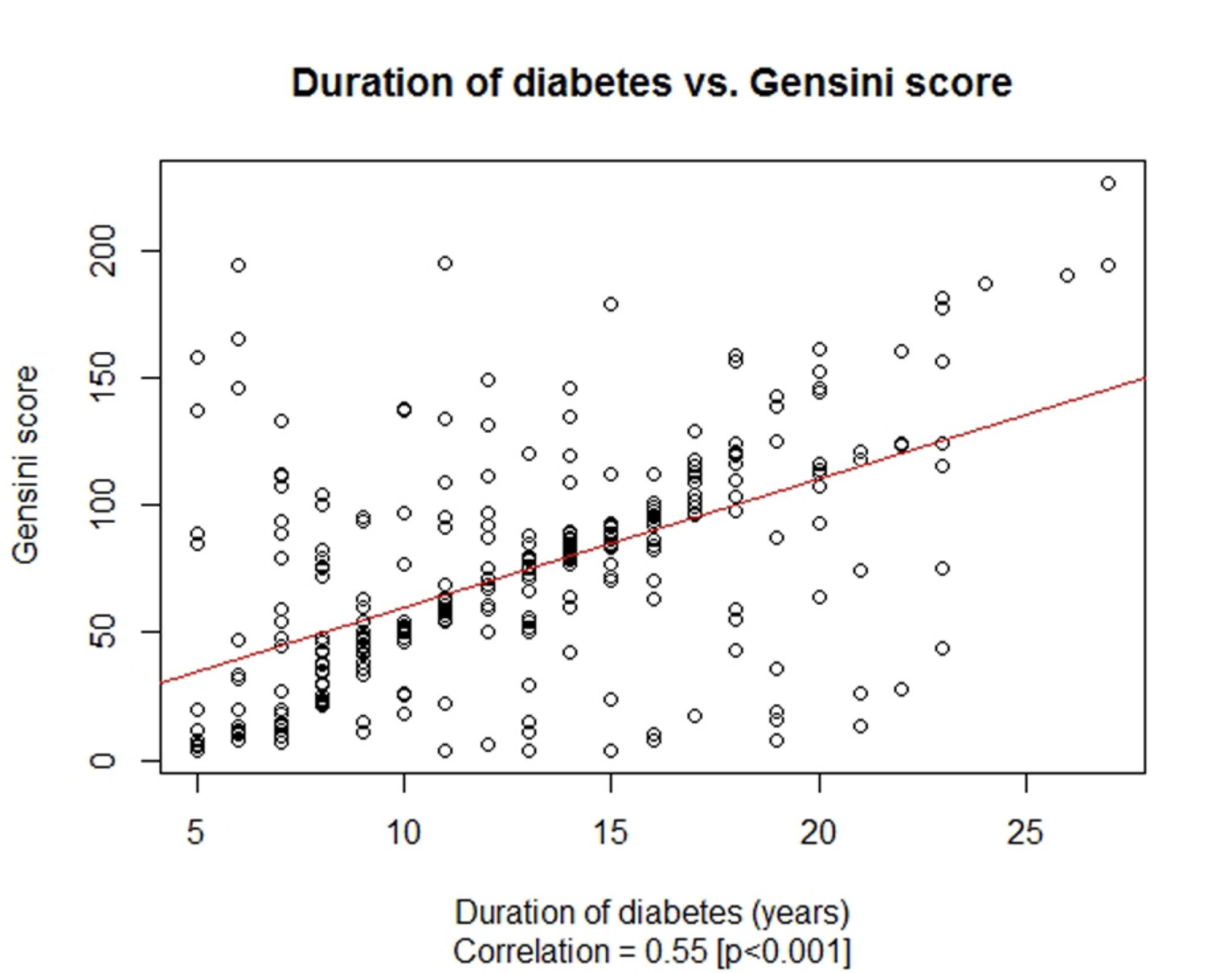
Correlation between the duration of diabetes mellitus (years) and Gensini score

Diabetic duration was found to be 12.46 ± 4.86 years, ranging between 5 to 27 years. In addition, the mean Gensini score was calculated to be 71.99 ± 44.12, ranging between 4 and 226. The hypothesis of normality of distribution for both the duration of diabetes and the Gensini score were rejected by Kolmogorov-Smirnov (KS) test p-values of <0.001. A significant positive correlation, 0.55 (p<0.001), was observed between the duration of diabetes and the Gensini scores. This relationship between the duration of diabetes and the Gensini scores has been presented in Figure 1.

**Figure 2:**
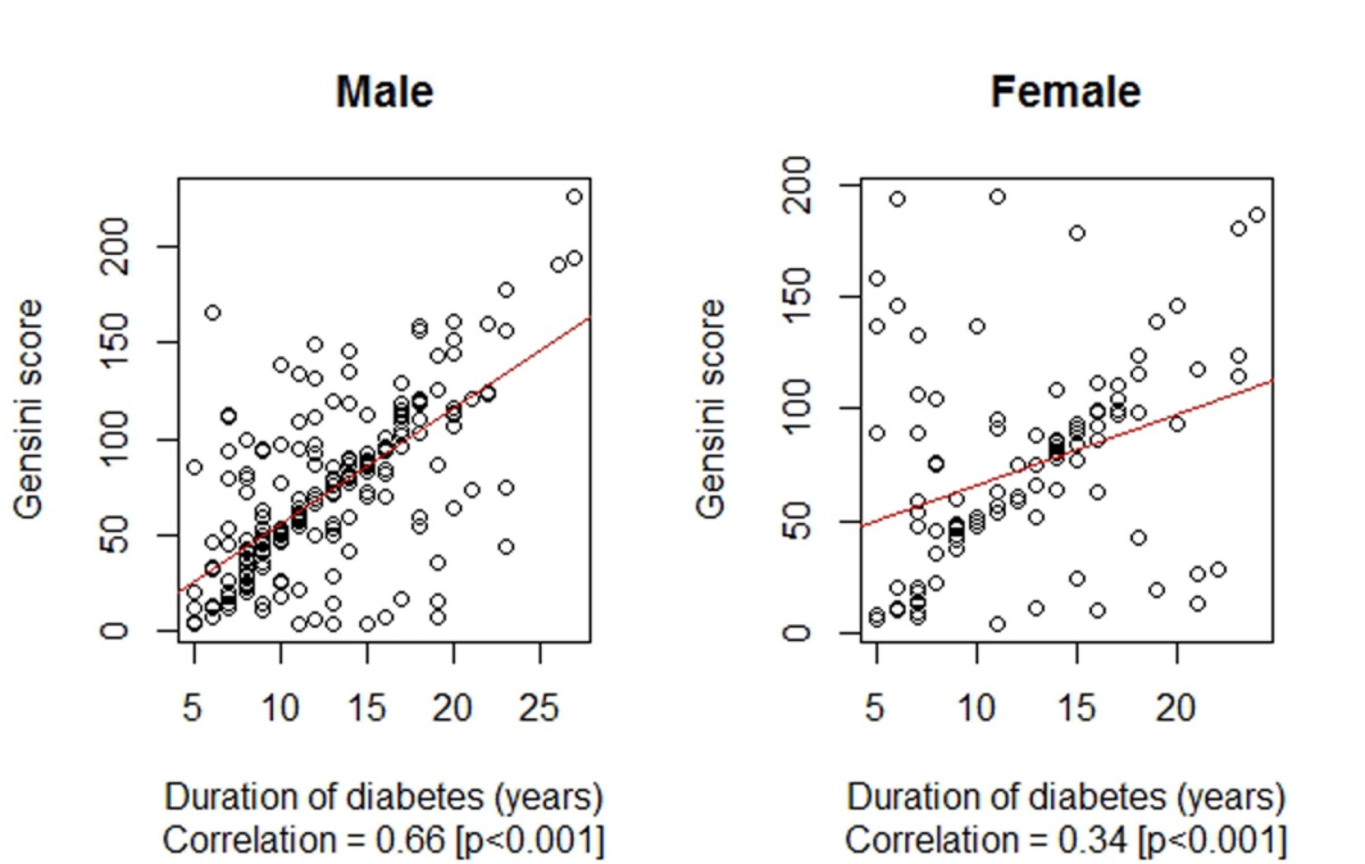
Correlation between the duration of diabetes mellitus (years) and Gensini score by gender

The differences in the duration of diabetes between the female and male subgroups was observed to be statistically insignificant at 12.25 ± 4.95 years vs. 12.56 ± 4.82 years, p = 0.547 respectively. Similarly, the difference in the Gensini scores between the female and male subgroups was observed to be statistically insignificant at 73.31 ± 45.71 vs. 71.36 ± 43.45, p = 0.806, respectively. Correlation between the duration of diabetes mellitus (years) and their Gensini score by gender are presented in Figure 2.

**Figure 3:**
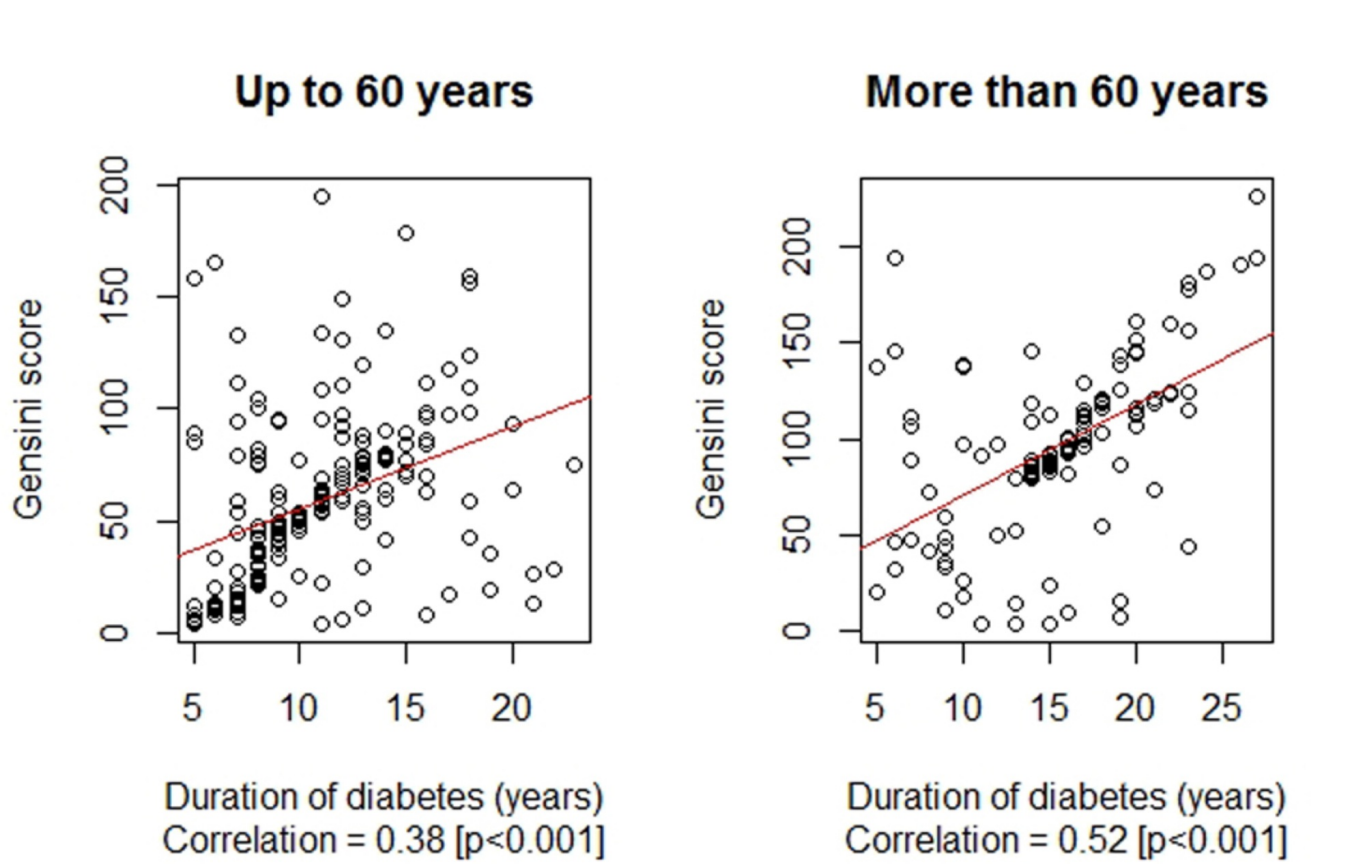
Correlation between the duration of diabetes mellitus (years) and Gensini score by age

The duration of diabetes and the Gensini scores were found to be much higher among the older age group (i.e., more than 60 years of age). The duration of diabetes was 10.74 ± 3.84 years vs. 15.51 ± 4.99 years, p < 0.001 for up to and more than 60 years of age, respectively. Similarly, the Gensini score was 58.36 ± 36.92 vs. 96.36 ± 45.40, p < 0.001 for up to and more than 60 years of age, respectively. The correlation between the duration of diabetes mellitus (years) and the Gensini score by age are presented in Figure 3.

**Figure 4:**
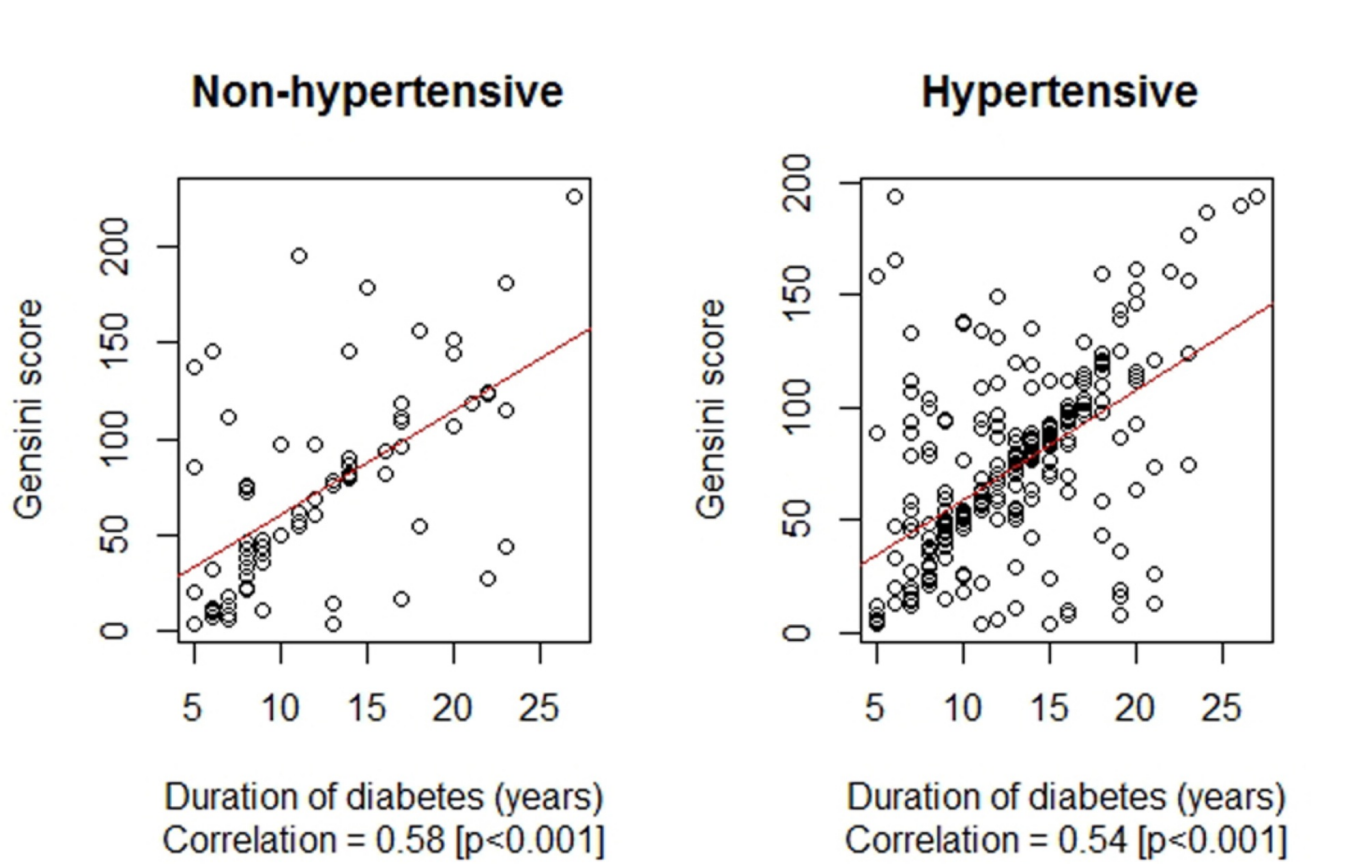
Correlation between the duration of diabetes mellitus (years) and Gensini score by hypertension status

The difference in the duration of diabetes between the non-hypertensive and hypertensive subgroups was observed to be statistically insignificant at 12.32 ± 5.57 years vs. 12.50 ± 4.60 years, p = 0.429, respectively. Similarly, the difference in the Gensini scores between the non-hypertensive and hypertensive subgroups was observed to be statistically insignificant at 72.96 ± 51.75 vs. 71.71 ± 41.78, p = 0.746, respectively. The correlation between the duration of diabetes mellitus (years) and the Gensini score by hypertension status are presented in Figure 4.

Similarly, baseline smoking status and obesity were found to have no statistically significant impact on the duration of diabetes and the Gensini score. The duration of diabetes mellitus (years) and the Gensini score by baseline smoking status and obesity are presented in Table 2.

**Table 2:** Duration of diabetes mellitus (years) and Gensini score by baseline smoking status and obesity p-values are based on the Mann-Whitney U test

Characteristics	Variable	Base (N)	Mean ± SD	p-value
Duration of diabetes (years)	Non Smokers	261	12.52 ± 4.89	0.699
	Smokers	60	12.18 ± 4.74	
Ginisini Score	Non Smokers	261	73.32 ± 44.67	0.243
	Smokers	60	66.18 ± 41.51	
Duration of diabetes (years)	Non-Obese	265	12.52 ± 4.87	0.699
	Obese	56	12.2 ± 4.84	
Ginisini Score	Non-Obese	265	73.39 ± 43.6	0.176
	Obese	56	65.36 ± 46.34	
p-values are based on the Mann-Whitney U test				

## Discussion

Diabetes mellitus (DM), especially type two, is firmly associated with the risk of cardiovascular disease (CVD) due to the multiple shared risk factors [17-18]. Epidemiologically, it is among the leading public health concerns of modern times, and it is attributed to an increased risk of cardiovascular morbidities and mortality [19]. Alongside its prognostic strength, diabetes is an important modifiable cardiovascular risk factor, and the duration of diabetes is associated with varying spectra of coronary artery disease [20]. Due to various lifestyle modifications and other causes, metabolic disorders are on the rise in developing countries such as Pakistan [21]. Therefore, in this study, we aim to assess the correlation between the duration of diabetes and the severity of coronary artery diseases as assessed by Gensini Score.

In our study group of 321 diabetic patients, the mean duration of diabetes was 12.46 ± 4.86 years, and the corresponding mean Gensini score was calculated to be 71.99 ± 44.12, with a significant positive correlation of 0.55 (p < 0.001) between the two variates. Both the duration of diabetes and the Gensini scores were statistically insignificant by gender; however, the strength of association between the two was relatively more strong among male patients compared to female patients, with correlation coefficients of 0.66 vs. 0.34. Also, the duration of diabetes and Gensini score were found to be much higher among older patients (more than 60 years of age), and the association was found to be stronger in older patients (more than 60 years of age) with a correlation coefficient of 0.52 vs. 0.38.

In our study, we found a positive correlation that is aligned with the findings of past studies [14,22-23]. In a study, two or three vessel disease was found to be more common (94.1%) among patients with a diabetic duration of more than 10 years [24]. A study conducted in our local population by Salem et al. [23] reported a correlation coefficient of 0.36 (p = 0.004) between the Gensini score and duration of diabetes. The correlation reported by Salem et al. [23] is relatively smaller than the correlation observed in our study. One potential reason for such discrepancy might be the effect of sample size differences between both studies.

One of the key limitations of this study is its cross-sectional nature along with single center coverage. Secondly, in this study, patients over 70 years of age were excluded from the study; therefore, the interaction of diabetes with CAD could not be found for the section of the population over 70 years of age. For the clinical utility of these findings, it needs to be further validated by multicenter large studies.

## Conclusions

A significant positive correlation between the Gensini score and the duration of diabetes mellitus was observed. This correlation is relatively stronger among males and older patients (more than 60 years of age). Given this positive relationship, not only the presence of diabetes but also the duration of diabetes should be considered for the risk stratification of patients.
